# Exploring the experience of Gamblers Anonymous meetings during COVID-19: a qualitative study

**DOI:** 10.1007/s12144-021-02089-5

**Published:** 2021-08-17

**Authors:** Katy L. Penfold, Jane Ogden

**Affiliations:** School of Psychology, University of Surrey, Guildford, GU2 7XH UK

**Keywords:** Gamblers Anonymous, Gambling, Mutual aid, Peer support, COVID-19, Gambling addiction

## Abstract

Whilst much research has explored the possible causes and consequences of gambling, Gamblers Anonymous (GA) − one of the most accessed forms of support for gamblers - has been largely overlooked and, to date, only a few studies have explored how members experience this programme. Core to GA is the social interaction between members. From March 2020, however, the COVID-19 pandemic forced GA to move their meetings online. The present qualitative study therefore explored how GA members experienced these online meetings in the absence of actual face to face interactions with others. Individual telephone or video call interviews (*n* = 21) were carried out with members of GA in the UK and analysed using Thematic Analysis. The results described three main themes: (1) ‘practicalities of GA in lockdown’, which highlighted the practical benefits of online meetings such as more opportunity to attend different meetings, which in turn expanded participants’ perspectives and social networks; (2) ‘the importance of relationships in GA’, reflecting strong and enduring social networks that were created, maintained, and strengthened by feelings of solidarity; and (3) ‘therapeutic elements of the meetings’, such as psychological contract making which helped participants to stay abstinent. Transcending these themes was a tension between individual versus group identity with interviewees reporting a shift to focusing more on their own needs rather than those of the group. Overall, whilst still providing a lifeline during COVID-19 and offering some practical benefits, the online GA meetings were not able to completely replicate the value individuals gained from face to face meetings. This transition also resulted in disruptions both to group dynamics and to individual interactions within each group, ultimately resulting in participants behaving more individualistically and less collectively than in face-to-face meetings.

## Introduction

The COVID-19 pandemic is a global public health emergency, which healthcare systems around the world were not sufficiently prepared for (e.g. Jatta et al., 2021). By January 24th 2021 there were roughly 98.2 million reported current cases worldwide with 2.1 million deaths confirmed (World Health Organisation, 2021). In conjunction with severe physical health implications, concerns about the impact on mental health have also been raised (Holmes et al., 2020). Though too early to understand the full psychological impact of the COVID-19 pandemic, previous research concerning other recent global pandemics has revealed a profound and broad spectrum ranging from sleep disturbances, shame, fear (of pain/dying/being separated from loved ones), anxiety, depression, trauma, panic to psychosis, dissociation and suicidal ideation (Hall et al., 2008; Müller, 2015; Tucci et al., 2017).

As with other countries around the world, since the start of the pandemic, the UK has seen strict ‘stay at home’ orders put into place. These measures had a profound impact on every part of life as suddenly individuals were required to fulfil all of their normal tasks at home, via the Internet. In some instances, this measures resulted in many advantages, such as working from home, which it has been argued that the advantages outweigh the disadvantages due to an increase in flexibility, productivity, efficiency, satisfaction and improvements in work/life balance (Beno, 2021). However, these restrictions also lead to concerns regarding the effect of these restrictions on a range of harmful behaviours such as problematic video gaming (King et al., 2020), Internet use (Sun et al., 2020), alcohol and substance use (Mallet et al., 2021), and gambling (Håkansson, 2020). This study focused on gambling during the COVID-19 pandemic and the impact of lockdown measures on Gamblers Anonymous (GA) meetings in the UK.

Research focusing on previous national crises illustrate a link with increased gambling behavior. For example, a study in Greece by Economou and colleagues (Economou et al., 2019) revealed an increase in problem gambling (especially among women) related to the financial crisis of 2008. Similarly, reporting on the results of three national gambling prevalence studies in Iceland, Olason and colleagues (Olason et al., 2015) revealed an association between the 2008 financial crisis and an increase in both gambling participation and problem gambling, especially online gambling in males. Thus, whilst research is currently limited, both the ‘stay at home’ order and the financial ramifications of the current COVID-19 pandemic could motivate people to gamble and increase gambling-related harms.

Problematic gambling is associated with a number of harms including substantial financial, psychological, physical and relationship decline (e.g. Battersby et al., 2006; Cunningham-Williams et al., 1998; Dowling et al., 2009; Ferland et al., 2008; Kalischuk et al., 2006). Across different countries and continents, problem gambling affects between 0.1% and 5.8% of the general population (Calado & Griffiths, 2016). In the UK, 0.7% of the general population (around half a million people) experience problem gambling, with 3.6% (a further two-and-a-half million people) at low or moderate risk (Sullivan, 2019).

Problem gambling is treated in several ways including pharmacotherapies, family-marital therapies, psychoanalytic/psychodynamic approaches, behavioural therapy, cognitive therapy, cognitive-behavioural therapy, and brief and motivational approaches. The most widely available treatment approach, however, is Gamblers Anonymous (GA; Petry, 2005) which is a nonprofessional, self-supporting, apolitical mutual aid fellowship.

Mutual aid fellowships tend not to give advice but opt for empathy and assistance. Fundamentally, mutual aid groups are free and open to all; the only requirement for membership is a desire for sobriety. However, GA is different from other mutual aid groups (such as Alcoholics Anonymous; AA) as it tends to place more of an emphasis on helping with financial difficulties associated with gambling problems (Ferentzy et al., 2006). The involvement of family members and social network is also emphasized (Ferentzy et al., 2010).

Most research on mutual aid groups has focussed on groups for alcohol and substance issues (such as Alcoholics Anonymous or Narcotics Anonymous), with research into Gamblers Anonymous only gaining momentum in the last few years. Furthermore, whilst several researchers have focussed on the clinical effectiveness of mutual aid groups (e.g. Kelly et al., 2020; Schuler et al., 2016), less is known about the underlying mechanisms involved. They are, however, frequently cited to provide social support, which has been demonstrated to facilitate both abstinence and recovery for various addictions (e.g. Best et al., 2016; Buckingham et al., 2013; Dingle et al., 2015).

In an attempt to understand the social processes involved in AA, Groh et al. (2008) performed a systematic review which examined the association between AA and social network variables. Their results revealed that involvement in AA facilitates positive change in the social support members receive; the social networks created through membership to AA were of great value to individuals’ recovery, and those who had more damaging and negative social support networks (those which supported drinking alcohol) prior to joining AA derived the most benefit from the meetings. Specifically, the review highlighted a link between AA involvement and quality of friendships, more friendship resources, greater social support, reduced support for alcohol consumption by friends, and increased support for abstaining from alcohol by friends. The results were further supported by qualitative findings which indicated that relationships made within AA provided more support, trust and respect than relationships which were made prior to, and outside of, involvement in AA. Furthermore, social support variables consistently mediated the impact of AA on individuals’ abstinence, suggesting that an underlying mechanism of the effectiveness of AA is social support.

Despite the popularity and accessibility of GA, little is known about the actual experiences of attendees, how they interact with the programme, or the underlying mechanisms involved. One study by McGrath et al. (2018) investigated involvement in GA among attendees, motives for attendance, and overall satisfaction with the program. They administered a series of self-report questionnaires to 512 patients presenting for treatment at a gambling outpatient facility in Sao Paulo, Brazil. Results revealed the number of days attended in the 30 days prior to the study was 5.5, with a range of 1 to 30. Individuals generally participated passively in the meetings; the most common activity engaged in was ‘listening to testimonials’ (with 86.2% of participants having engaged in this activity). Other activities engaged in included ‘performed minor service (organizing the room, preparing coffee, etc.)’ (9.2% of participants); ‘other’ (6.2%); ‘acted as a sponsor/helper or guided a peer in distress’ (2.3%); ‘coordinated meetings’ (2.3%); ‘assisted beginners or gave telephone guidance’ (2.3%). The least common was ‘assisted the coordinator or treasurer’ (0.8%). Interestingly, they also found that those who reported never having given a testimonial scored significantly higher on a measure of gambling symptom severity than those who provided at least one testimonial. Most reported having a sponsor (57.7%), though in general participants reported having little (33.3%) or no (32%) contact with their sponsor. Only 24% reported having regular contact with their sponsor. Not having contact with a sponsor lead to significantly higher gambling symptom severity. The most common motivation for attendance was to be reminded of the consequences of relapse (45.9% of participants reported being motivated by this). Other motivations included being in contact with people who can provide understanding of their problem (28.1%), to strengthen commitment to abstinence (21.5%), to vent or relieve pressure (11.9%); to learn how to deal with financial, personal, and legal problems (8.9%); and to catch up with friends and meeting people (2.2%). The results of this study offer a much-needed insight into GA, providing both a descriptive and analytical account, however the sample consisted of problem gamblers presenting for outpatient treatment at a psychiatric unit in Brazil, thus the results may not reflect the experiences of those for whom GA is the only source of support, or of GA members in the UK. Furthermore, using questionnaires limited participant responses to a set of pre-defined answers, and cannot, therefore, provide a rich and in-depth understanding of GA as experienced by its members.

Due to the COVID-19 social restrictions, from March 2020 face-to-face Gamblers Anonymous meetings were prohibited. This left members vulnerable to relapse at a time when they were potentially at an increased risk of gambling harm (Economou et al., 2019; Olason et al., 2015). In response, GA established online meetings within a couple of weeks which were available for members. This changed the experience of GA for members as meetings were held over Zoom.

### The Current Study

Whilst much research has explored possible causes and consequences of gambling, Gamblers Anonymous - one of the most accessed forms of support for gamblers - has been largely overlooked and to date, only a few studies have explored how members experience this programme. Furthermore, to date no research has explored how mutual aid groups like Gamblers Anonymous have been affected by the COVID-19 pandemic. Therefore, whilst little was known about the experience of GA prior to lockdown, less still is known about what that experience is like during a pandemic; for instance, if/how people have found a way to continue attending, whether alternative methods are viable, how they compare to traditional meetings and, crucially, whether people are still receiving adequate support.

The current study therefore aimed to address this gap in the literature by providing an insight into members’ experiences of attending GA meetings and how they feel this has been impacted upon by the COVID-19 pandemic and the move to online meetings. It is hoped that by providing this insight, Gamblers Anonymous and other service providers will be able to make more informed choices about how to support their users, should we face another global crisis. This knowledge will also help organisations like Gamblers Anonymous – and other mutual aid groups – to understand their members’ needs, ultimately optimizing the support they can offer.

First, the method used for this study will be presented, followed by a synthesized report of the results, a discussion including how these results sit within the existing academic literature, and the strengths and limitations of this research. Finally, the conclusions drawn from this research are presented.

## Method

### Design

A qualitative design was used to obtain detailed data sufficient for a rich and in-depth understanding of GA members experiences of GA during COVID-19. Data was collected using semi-structured telephone or video call interviews, which were analysed using Thematic Analysis (Braun & Clarke, 2006).

#### Participants

Participants (*n* = 21) were 18 men and 3 women who were active members of Gamblers Anonymous across the UK. Participants were asked to give some details about themselves and their involvement with Gamblers Anonymous though less than half (*n* = 9) chose to do so. Of those that responded, most were White British (*n* = 7), with the remaining 2 identifying their ethnicity as Christian. All but one participant reported using video communication to attend meetings, with the other using audio-only teleconferencing software.

#### Procedure

Purposive sampling was used for recruitment. Emails were sent to chapters of GA across the UK explaining the study and inviting people to take part, should they wish to do so. Email addresses for each chapter of GA were obtained from the GA website (https://www.gamblersanonymous.org.uk/find-a-meeting). Participants were included if they were an adult over the age of 18 years, were active members of GA, had experience of GA meetings during the COVID-19 lockdown period in the UK and were able to speak and understand English.

Participants were interviewed predominantly by telephone (*n* = 20), though one was interviewed via Zoom (video call software). An interview schedule comprising the following seven open-ended questions was used: 1. “Can you tell me about your experiences with Gamblers Anonymous?”; 2. “Can you tell me about your experiences with online Gamblers Anonymous meetings?”; 3. “How do you find the online meetings?”; 4. “How often do you take part in online Gamblers Anonymous meetings?”; 5. “How are online meetings different from face-to-face?”; 6. “Do you find the online meetings helpful? (In which ways do you find the online meetings helpful?)”; 7. “Are there any aspects of the online meetings that you don’t like?”. Asking questions about GA pre-lockdown provided context for the questions regarding the experience of GA during COVID-19, helping the researcher to gain a clear insight into what the online meetings are like and how the two experiences compare. The interviews lasted between 30 and 80 min. Data was collected between June and August 2020.

The interviews were audio recorded and transcribed verbatim. During this process participants were assigned pseudonyms to maintain their anonymity.

#### Data Analysis and Theoretical Approach

Thematic Analysis (TA; Braun & Clarke, 2006) was identified as the most appropriate analytic approach as is allows flexible, data-driven analysis, rather than one tied to a specific theoretical framework, which encourages a structured approach to analysis (Nowell et al., 2017). Thematic analysis allows for a good overview and summary of a large amount of data (which can be generated by under-researched topics) and has the potential to generate “unexpected insights” (Braun & Clarke, 2006). Thus, the analysis is informed by, rather than being driven by existing theory. Furthermore, due to the nature of the research topic there was little previous research; this approached allowed the analysis to be inductive, reflecting the lived experiences of the participants of this study.

The analysis was conducted with a critical realist epistemological perspective (Bhaskar, 1975). This allowed the researcher to accept that the data reflected the objective realities of the participants and conduct the analysis as such, however it was also acknowledged that the reality of each participant sits within a cultural, societal, and historical context.

The transcripts were analysed in six stages, in accordance with the Thematic Analysis guidelines as described by Braun and Clarke (2006), which are as follows: (1) familiarisation of data through repeated reading. Anything interesting or significant was noted, and potential themes were recorded in a research diary, (2), initial coding using NVIVO (qualitative data analysis computer software); these codes identified features of the data which the researcher felt to be important to the research question, (3), searching for emerging themes which were noted separately, (4) reviewing themes by returning to the original data set and comparing the themes against it, (5), defining and naming themes, and (6) producing the report, including verbatim examples taken from the transcripts. This was an interactive process involving ongoing discussions with the research team.

#### Compliance with Ethical Standards

Ethical approval for this research was obtained from the University Research Integrity and Governance Office (RIGO). Participants signed a consent form which included details about: the background and purpose of the study; why they were invited to take part; their participation being entirely voluntary; what would be required of them; assurances of anonymity, confidentiality, and data protection; and risks, disadvantages, and benefits of taking part. Additionally, participants gave their consent for the interviews to be recorded.

## Results

Through analysis and repeated coding three main themes were developed which included several related subthemes. The themes were separated into different levels, with three main themes and a transcending theme. An overview of the themes is presented below, followed by a detailed analysis of the themes and their related subthemes, demonstrated with verbatim quotes from the transcripts.

### Overview of Themes

**Diagram 1:**
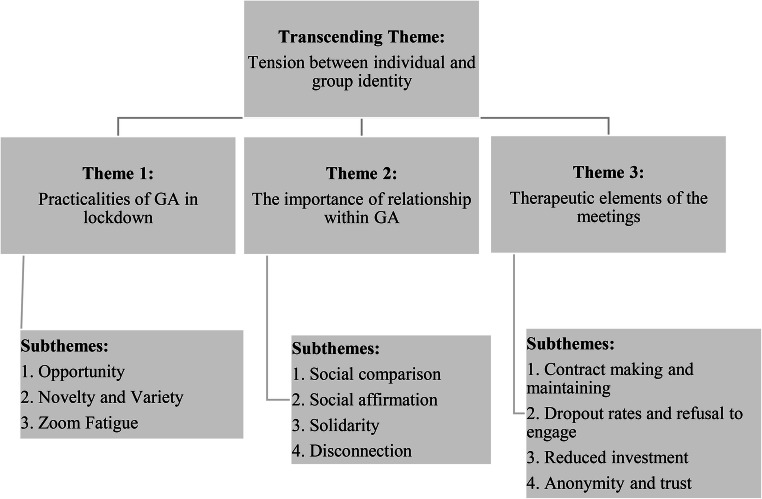
Hierarchy of Themes

Participants’ responses centered around three themes i) ‘the practicalities of GA in lockdown’; ii) ‘the importance of relationships within GA’ and iii) ‘therapeutic elements of the meetings’. Transcending these themes was a tension between individual and group identity which described the complex nature of, and tension between, individual and group identity within GA, and how identity affected the overall experience of participants. These themes and their subthemes will be discussed below and illustrated with exemplar quotes (Diagram 1).

### Theme 1: Practicalities of GA in Lockdown

This theme has three subthemes: opportunity, novelty and variety, and Zoom fatigue.

#### Opportunity

This subtheme encompasses the various and unique opportunities that attending GA in an online environment presented. Several practical benefits were identified, most notably due to the removal of geographical boundaries. Whilst some participants chose to continue solely attending their (online) local meeting, the transition to Zoom meetings allowed people to have more choice over which meetings to attend. As such, many participants chose to attend meetings further afield, including both nationally and internationally:

*“I’ve been joining 2, 3, sometimes 4 meetings a week. The great thing is, with these Zoom meetings, you can join meetings all over the world … You know, you can join one in Hawaii one day, Sydney the next day, you know, America, all over.” (Sebastian).*

This quotation shows that Sebastian has increased the number of meetings he attends, embracing ‘travelling’ “all over the world” to attend them. This aspect may have been particularly appealing because at the height of lockdown, individuals were unable to leave their homes for more than an hour a day. The ability to not only continue attending meetings but gain some sense of freedom, even if in a virtual way, may have been cathartic for people.

People were also able to attend different *types* of meetings. For example, in the UK, whilst all meetings are based on the 12-step principals, only a small proportion of the meetings are classed as ‘steps meetings’ which focus on working through each of the 12 steps of GA. For many, the ability to attend different meetings meant they were able to specifically choose these meetings and found more value in them than their regular meetings. For example, Brian describes how the decision and ability to log onto the ‘steps meetings’ on Zoom changed his approach to his recovery, and strengthened resolve after relapse:

“*...the only reason I’ve changed this time is because I’ve gone onto the steps meeting on Zoom. If I’m honest, and that’s what the GA programme is all about, and that’s the message I’m hearing on the Zoom meeting is…get on the Steps meetings. Get on the steps.” (Brian).*

Brian specifically states that if he hadn’t “gone onto the steps meetings” - which were only made available to him because of lockdown - then he believes he would have relapsed.

The above quotations demonstrate how the transition to Internet-based meetings not only allowed participation in the programme to continue but presented unique benefits which resulted in positive outcomes. In this way, the participants of this study demonstrated resilience in the face of adversity, and managed to adapt quickly to the new scenario.

#### Novelty and Variety

The opportunities presented created a sense of novelty and variety, resulting in a ‘fun’ and positive experience. For example, Brian discussed an excitement from GA members from all over the world to participate in his local meeting, which lead to a positive and enjoyable experience for him. He said:

*“On Saturday there was about 10 or 12 people on it, and again we had a couple of foreign people on it and stuff like that, and it’s great, and I think, just where I’m from … people come on because of that, Liverpool. There was one fella with a Liverpool top on on Saturday all made up. But people see Liverpool, they go “oh, yeah”. So, I think that meeting is really going to grow and it’s only going to get better.” (Brian).*

Brian speaks with a sense of pride about his hometown and alludes to other people being excited to attend the Liverpool meeting, which he enjoys. He also expects that because of this novelty the group is likely to attract more new members which will subsequently improve the quality of his group. Participants enjoyed the experience of new members (or being a new member to other groups) because they were able to gain different perspectives which allowed them to think about their own recovery in new and different ways:

*“… I can’t just get on a plane and go “right, I’m off to GA in LA today”. So, that’s opened new doors and it all helps to recovery because you get that many different perspectives if you speak to people from similar situations but with their own story and it’s all useful.” (Fergus).*

Fergus highlights the new opportunities presented, stating that the online meetings have “opened new doors” for him due to the new perspectives he was able to gain through attending meetings internationally. His suggestion that other members have similarly attended different meetings implies that he feels this positive experience is not unique to him.

#### Zoom Fatigue

As all other aspects of people’s lives (work/leisure) moved online during lockdown, it is perhaps unsurprising that participants struggled with Zoom fatigue. It was commonly reported that participants would be required to spend their entire days at their screens, sometimes in conditions which were unsuitable for working, which resulted in feelings of apathy and demotivation towards the GA meetings.

“*it’s easier to actually attend because you don’t have to go anywhere but at the same time, if you’ve been sat at a desk or a makeshift desk and on Zoom meetings all day and you’re a bit frazzled then I can understand why at 8 o’clock at night the last thing you really want to be doing is getting on another Zoom meeting.” (Fergus).*

This quotation shows that, despite Fergus acknowledging the increased physical ease of attending online meetings, he struggled with the monotony and cognitive effort of being on Zoom all day, leading to apathy towards GA because it would mean more time spent on Zoom. The Zoom fatigue was not sufficient to stop people attending the meetings all together, however, and the perception of the importance of the meetings remained:

*“Zoom is better than nothing. That’s the long and short of it”. (George).*

This quotation concisely encapsulates the feeling that despite not being on par with the physical meetings, participants felt better off having online meetings than none. Whilst some participants felt the online meetings were somewhat substandard, but acknowledged they were “better than nothing”, others felt much more positively towards them, going as far as to say that if they did not have the online meetings, they would have relapsed.

### Theme 2: The Importance of Relationships within GA

Through GA, participants developed social networks which were integral to their experience and supported their wellbeing. These networks fulfilled various functions, which centre around four subthemes: social comparison, social affirmation, solidarity, and disconnection.

#### Social Comparison

Participants used social comparison through listening to the negative experiences of others to reinforce the importance of continuing to attend meetings, as Sebastian described:

*“you think you can do it on your own and stuff like that but unfortunately you sort-of forget how important the meetings are, and going to the meetings, and hearing the horror stories sometimes really helps you”. (Sebastian).*

The use of the words “you think you can do it on your own” suggests that the meetings provide feelings of togetherness and support, without which recovery would be impossible. The term “horror story” frequently occurred throughout all the transcripts, which evokes a sense of panic and terror when thinking about their gambling experiences prior to attending GA.

“*What I like about GA is when the new members turn up and you hear their horror stories, and then that makes it really kind-of raw and brings back the memories … that’s what it was that always worked for me. Hearing – I don’t want to be there – and it reminds me if I take my eye off the ball that’s exactly where I’m going to be. (Rick).*

For Rick, hearing the “horror stories” of other peoples’ experiences serves firstly as a kind of benchmark of where they do not want to be, but also as a reminder about previous negative experiences, which strengthen his focus and determination about his own recovery. Participants also used social comparison in a more positive way, finding inspiration from hearing about other people’s positive recovery stories, which helped them feel hopeful about their own recovery. The data conveyed a sense that, by comparing themselves to others, participants were able to understand their own recovery better, and understand the context of it; that there were others like them.

#### Social Affirmation

The meetings helped participants to achieve a positive self-concept, which was identified as a key motivator for continued attendance. The way the meetings provided social affirmation were twofold. Firstly, they provided a place for people to share their sobriety which fostered feelings of pride:

*“When there’s someone giving their therapy, I think there’s kind-of this element of pride almost of like, you know, “I haven’t gambled in 18 months or I haven’t gambled in 2 years therefore I’ve got great things to say about this. Oh, I remember when I was there”. (Grant).*

Grant describes the process of listening to other members experiences resulting in feeling pride about his own recovery achievements. Grant also feels value in being able to speak about his experiences with those who are less far along their recovery journey. This process of reflection connotes a sense of mental solidity about his recovery which serves as a reminder that he does indeed have something to be proud of. Secondly, participants received social affirmation for attending meetings, in the form of group praise and support.

*“I’ve come off the wagon…squandered…chosen to gamble many times in those eight years. But I’ve always attended back to GA … it’s just nice because you go back and even though it’s a lower point to go, they’re obviously very understanding…and erm, they are…congratulate you for being honest and getting back into…obviously, getting back to, erm, on the road to recovery.” (Rick).*

Rick describes feelings of unconditional acceptance from the group, despite having gambled on more than one occasion during his time as a member of GA. Even though each time he has gambled he has perceived himself to be going back to the group at a “lower point”, he has felt that the group have met him with praise for returning.

#### Solidarity

These social support networks may be created, maintained, and strengthened by the feelings of solidarity the peer support group environment facilitates.

*“As a team on a Sunday evening we all have each other’s backs, and we all understand what’s required of the meeting, everybody just has the ability to listen without having a self-opinionated point because everybody just wants everybody to stop gambling.” (Billie)*

Billie describes the people at the meeting as being a “team” which shows that, beyond being acquaintances at a support group, she considers herself and the other members a cohesive group, acknowledging shared goals (to stop gambling). These common goals coupled with a perceived lack of judgement from the group implies strong social relationships, further demonstrated by the phrase “have each other’s backs” which is indicative of a sense of loyalty and togetherness.

This however has been affected by the move online, both directly (because of being separated physically when attending meetings) and indirectly (because many members decided not to participate in the online meetings affecting the group dynamics). Because not all the members of the meeting were willing to continue attending the meetings via the online platform, this caused some members to reject them from the group, for example:

*“… I don’t want you to be hearing about what’s going on in my GA meeting if you’re not even part of that community, like piss off, like you shouldn’t be there. You shouldn’t be privy to all this information because you no longer are part of the confidentiality, you shouldn’t be part of that community.” (Grant).*

It is clear Grant felt quite strongly that because of the unwillingness of some to attend the virtual meetings, they should no longer have access to the privileges being part of the group (such as being included as part of the community) affords. In this instance, the unwillingness to engage with video calls has been interpreted as an unwillingness to engage with the group in general, removing the element of solidarity. Without feelings of solidarity, Grant feels mistrust towards them as they are “no longer part of the confidentiality”.

#### Disconnection

This subtheme described how online meetings were unable to sufficiently encapsulate the feelings of emotional connection which is associated with physical presence. Participants were unable to describe specifically why they felt this way, though it was clear that the video calls could not sufficiently recreate the social experience that people felt when they were physically together. As a result, the experience of the meetings was tarnished.

*“I really can’t put my finger on it, but it’s just…there’s just something about that room. When we’re all sat in the room and we’re face-to-face discussing things it’s a lot easier.” (Don).*

“*I do feel like they’re lacking in something, but I also can’t quite put my finger on what it is apart from the obvious you know, of the actual people in the room kind-of feeling. (Craig).*

Don and Craig both identify that not being physically together changed the dynamics of the meetings, though were unable to define why. It was interesting to note that slight variations on these quotations came up in almost all interviews which suggests that there is some unseen or undefinable mental connection that is related to physical presence or nonverbal communication which cannot be captured via a video call.

“…*there is a lack of, sort-of, humanity is not the right word for it, but the fact that you’re sat looking at a screen or listening to other people rather than sitting in a room with other people…there’s something that’s a bit…dehumanizing about that.” (Fergus).*

Removing the physical presences and nonverbal communication from the meetings also has the effect of “dehumanizing” them; participants can see and speak to each other though doing so via a computer or phone does not provide them desired (or expected) social experience. The use of this word highlights that participants were acutely aware of the inanimate nature of phones and laptops, despite them facilitating some social interaction. That is, the social element does not permeate their experience sufficiently for them to forget they are on a computer at home.

### Theme 3: Therapeutic Elements of the Meetings

This theme outlined the features of the meetings which impacted participants’ sense of wellbeing (and were commonly related to more formal therapy). The subthemes are: contract making and maintaining, dropout rates and refusal to engage, reduced investment and anonymity and trust.

#### Contract Making and Maintaining

Behavioural contracting is a therapeutic technique in which an agreement is reached between the therapist and client stating goals and the consequences of meeting (or failing to meet) those goals. The participants in this study seemed to use meeting attendance as a kind of behavioural contract strategy; the goal was to remain sober from gambling, the reward for this behaviour was positive affirmation (through pride and praise from the group)*.* In fact, continued meeting attendance was perceived as the single most important factor for continued recovery, with participants attributing any ‘slips’ to ceasing attending.

*“The only time I really gamble is when I’ve missed meetings … I’ve gambled because I’ve missed the meetings. Very much so.” (Rick)*

*“Knowing what I know about the history of GA and the history of people wrecking their lives…slips are almost certain if you completely stop going to GA.” (Grant).*

These quotations show it is not only a perception that missing meetings results in a return to gambling (as with Grant) but is a real lived experience (as with Rick). In this way the meetings can be understood as psychological contract maintenance; attending each meeting renews this contract. Understandably then, having the physical meetings taken away due to lockdown caused a great deal of anxiety and fear.

*“…when suddenly all the meetings shut in March, I was quite worried about what are we going to do? How are we going to get our medicine? Fortunately, you know, after a week…a couple of weeks, these Zoom meetings started popping up and then it sort-of took off. So, that was a big relief for me because, you know, going 3, 6 months without a meeting could be very dangerous…” (Sebastian)*

This quotation demonstrates Sebastian’s worry that GA would be unable to adequately respond to the COVID-19 lockdown situation, and the meetings would simply cease. His use of the words “how are we going to get our medicine” implies that meetings are the only thing maintaining his wellbeing. Describing the Zoom meetings as a “relief” implies that the online meetings were able to provide support (or “medicine”) sufficient to keep him well (and abstinent). However, not all members felt this way. In several interviews, participants spoke about members who did not embrace the change:

*“I got really upset I nearly cried on a phone call with him once because I was like…we’re two and a half months into this lockdown and people haven’t been once, like what the fuck is going on here? People are…some of these people have been quit 10-15 years and I’ve seen them passionately and emphatically say “never miss a meeting. Meetings are our medicine. You cannot miss a meeting. I’ve been coming every week for 15 years, 10 years”, and then all of a sudden because the format changes, they’re being video calling over a laptop or an iPad or a phone, they’re like “yeah, I don’t like this. I’m not going to come anymore”, and it just didn’t make any sense, and I got really upset about it.” (Grant).*

Grant forcefully describes how important he perceives the meetings to be, and the extreme consequences he believes those who have not joined the online meetings will suffer. There is a real sense of desperation which again highlights how important GA meetings are to members. Grant also indicates that long-term contract maintenance is vital (even for those who have remained abstinent for many years). Participants recognised both that it was of the utmost importance for them to attend, and for others to attend. By speaking about this contract maintaining so emphatically gave the impression that this was the single strongest driver in motivation to attend GA.

#### Dropout Rates and Refusal to Engage

An immediate impact of GA meetings moving online was a significant dropout rate, mostly due to members refusing to engage with the new format.

*“I would say that there is…erm…75% of the members that go on Wednesday I’ve never seen online – 3 quarters. And that’s since March.” (Grant).*

The above quotation shows a significant dropout rate from Grant’s group. This commonly occurred throughout the transcripts. Given the importance of contract making and maintaining, this was alarming to participants:

*“Fifteen weeks is a long time not to have a meeting. And I do know, even a lot of other members, I’ve heard, who’ve been off a long time, wouldn’t go on Zoom, which is crazy, absolute madness, but yeah. Long-standing members refused to do it and I’m thinking what is going on?” (Harry).*

Harry describes how even well-established members declined to participant in the online meetings. The quotation again conveys a sense of desperation on behalf of the other members. The use of the words “crazy” and “madness” imply that it is so incomprehensible and extreme they must not be mentally well.

#### Reduced Investment

Participants felt prone to distraction in the online meetings, which affected how invested they were. Participant’s life routines were disrupted, and the way time was experienced changed from clearly defined ‘chunks’ (i.e., commuting/work/free time) to having much more blurred lines between activities. This in turn affected how they felt about the meetings preemptively, and their mental state during them.

*So, one of the things that I’ve found difficult is potentially people chairing them and not taking into account this is a virtual environment; people are at home in their own time; they’ve not specifically come out. They’re not as invested.” (Grant).*

Grant describes the absence of intention during the meetings because people are “on their own time”, suggesting the absence of any allotted time for GA within the home environment. As such, people are paying less attention, making it difficult for the chair of the meeting to ensure the meeting runs smoothly and members are kept engaged. The physical element of the meetings also seemed to somehow mentally oblige people to participate and engage.

*“…it’s definitely easier to tap out of it because if you don’t like what you see you can just close the screen on your computer, can’t you. Whereas the physical meeting, it’s a real life-changing moment for people, to come through those doors and sit down and say out loud “I’ve got a problem”.” (Craig).*

Craig suggests that the social convention which would prevent a person from standing up and walking out of a physical meeting no longer exists in the online environment (“if you don’t like what you see you can just close your computer, can’t you”). This also suggests that there are parts of the meetings which are not enjoyable or are uncomfortable, but social convention ensures members remain engaged for the full duration. Indeed, it was obvious that some participants clearly no longer felt obligated by social rules at all, and they became more self-seeking towards the meetings than they perhaps would have been in a physical meeting.

*“I’ll be walking the baby, and I’ll just jump on a meeting. I just want to listen. I’m just walking the baby, yeah, not a problem.” (Brian).*

Brian describes enjoying the ability to join a meeting when he is doing other activities such as going for a walk. He also describes how his intentions are just to listen, rather than contribute to the meeting in any way. This is beneficial for him because he does not experience any disruption to his life by having to attend the meeting but contrasts with the subtheme of *solidarity* as, certainly on this occasion, he is not concerned with offering anything back to the group.

#### Anonymity and Trust

Participants described how the transition to online meetings jeopardised the core principle of anonymity. Zoom was seen to pose two potential threats. Firstly, participants were worried about privacy in their own and other members homes.

*“The anonymity is a big thing for me … I just feel like you don’t have that in a Zoom meeting because you never know if your husband is in the background listening.” (Grant).*

Grant describes feeling anxious about the potential for family members to be in in the room and able to overhear the meetings. There were also concerns - and mistrust - around new members joining the meetings. Specifically, there was an issue around whether members should have to have their video on or not. Because GA is decentralised, and individual meetings run under their own volition, different groups adopted different stances towards camera use. Some groups felt their anonymity was preserved best by having their cameras off (so that other members’ families would not be able to see them). Some felt the cameras should be on all the time (to ‘level the playing field’). Casey said:

“*When we issue guidelines on online meetings, we were of the opinion that to protect anonymity everyone should have their video on so you could see that they were on their own, you could see who they were, you could see what they were doing. GA South Africa have taken the opposite approach, and they’ve actually said to protect anonymity everyone should have their cameras off. So, I think it’s important to acknowledge that there does seem to be a difference of opinion here. My view is that if you go to a meeting, people see you. Anonymity is not about what you look like, and so I think everyone should have their cameras on.” (Casey).*

### Transcending Theme: Tension between Individual Vs. Group Identity

Participants therefore described their experiences of GA online meetings during the pandemic in terms of three themes relating to the practicalities of GA in lockdown, the importance of relationships in GA, and therapeutic elements of the meetings. Transcending these themes was a tension between individual and group identity. In particular, whilst participants felt that the online meetings provided an environment which was able to nurture togetherness and collectivity (through facilitating a variety of social functions), the practical benefits they described were mostly individualistic (such as the ability to attend meetings for novelty and variety), and it was clear that group dynamics were disrupted by the absence of physical connection. When speaking broadly about their experiences of GA, participants revealed a real sense of solidarity, camaraderie, and group cohesion. Beyond this, though, participants felt duty-bound to help other gamblers and ‘give back’ to the group (a process they describe as ‘service’).

*“I’ve started doing a lot more service and trying to basically give back what I’ve got out of GA, and things have started to look up.” (Casey).*

Casey describes how ‘giving back’ has had a positive impact on his life. This quotation shows a complex situation whereby the goal is individual (ultimately each member of GA is there to facilitate their own recovery), but the means to achieving the goal are linked with group service. The extent to which people felt this intrinsic link differed, though it was present throughout all interviews. Because of this strong sense of group identity, and because of the decentralised nature of GA, the groups in general appeared to be strong, equal, supportive, and cohesive. The group dynamics were however changed in various ways by the transition to online meetings. Notably, the equality and mutuality of the meetings was affected by disrupted social norms.

*“…they wouldn’t do it if you was in a face-to-face meeting … I don’t think they’d just get up and walk out because I don’t think they’d be welcome back, but you can see that the same people will stop the video, they’re on mute so you don’t know if they’re there or not. Sometimes you just feel like saying “are you there? Are you listening?” (Martin).*

This quotation demonstrates that Zoom seemed to legitimise selfishness and self-serving behaviour. Usually (in physical meetings), each member can see that each other member is present and attentive. However, as Martin highlights, lack of consistency around camera use allowed people to pick and choose when they had their cameras on and when they had them off, disrupting the balance of the meetings. Several participants also described enjoying the ability to speak when they wanted to and leave the meeting when they had finished – something which would usually be considered rude.

*“Zoom is better for me. If I’m bored of it, I can just leave the meeting. I can go to the meeting, say my bit, listen to the advice and then if it is dragging on, I can just leave. If I’m not getting anything from it, why should I stay?” (Brian).*

By contrast to the principle of group unity, this quotation shows a very individualistic approach to the online meetings. Brian explicitly states that he is willing (and does) leave meetings if he feels he is not getting anything personally out of them. He clearly does feel as though he is getting something from speaking himself but sees no value in staying for other members. This suggests that the desire to perform ‘service’ and ‘give back’ to the group might, at least in part, be the result of pressure to conform to social norms rather than arising from a genuine motivation to serve the group; Zoom has provided a way for people to avoid the repercussions of breaking the social norms (disapproval of or even rejection from the group) which some participants gladly utilised, taking only the bits of the meetings they wanted. Thus, while the online meetings have certainly provided people with a viable source of support, moving GA meetings online appears to have changed the model of the programme from being a collectivist to more consumerist model.

## Discussion

This study explored the experience of attending Gamblers Anonymous meetings during the COVID-19 pandemic. The analyses described three themes relating to the practicalities of GA in lockdown, the importance of relationships in GA and the therapeutic elements of the meetings, which find reflection in previous research. For example, reflecting the work of (Beno, 2021), the Zoom meetings presented practical benefits such as more opportunity to attend different meetings and expanded participants’ social networks, which has been linked to more positive programme outcomes (Groh et al., 2008). Furthermore, the participants of this study reported that new perspectives gained by broadening their social networks and relationships with others and described strong and enduring social networks, which were created, maintained, and strengthened by the feelings of solidarity the peer support group environment facilitates. This was deemed to have had a positive effect on their recovery in line with research highlighting the social support mutual aid groups provide as being fundamental to the wellbeing of their members (Best et al., 2016; Buckingham et al., 2013; Dingle et al., 2015; Groh et al., 2008). Likewise participants highlighted several therapeutic functions of the meetings, including psychological contract making and maintaining which helped them stay abstinent; this appeared to be a strong motivator for attendance, also reflecting previous research in this area (Groh et al., 2008; McGrath et al., 2018). Participants did, however, find their investment in the meetings was reduced and there were anxieties about anonymity and trust on Zoom.

Transcending these themes was a tension between individual and group identity with participants highlighting a balance between part of a group experience whilst still finding benefit for themselves. For example, the Zoom meetings were still able to encapsulate many aspects of group experiences at least to some degree; social comparison continued to happen; they were still able to derive social affirmation; they still felt as though their social support networks remained intact, and they still experienced feelings of solidarity and togetherness with their groups. Zoom was however unable to sufficiently replicate the human connection felt at face-to-face meetings, which had a profound effect on group dynamics, and the level of social interaction within each group. For example, participants took value from giving testimonials, however, did not always feel it necessary or valuable to stay and listen to others’ testimonials. Interestingly, previous research has shown that most members of GA are engaged in the programme passively, with listening to testimonials being the most popular activity (McGrath et al., 2018).

The results of this study suggest that some members of GA might be passively engaged not because they are motivated to listen, but because social norms demand it; Zoom appeared to create a barrier to the human connection which provided a way for people to avoid the repercussions of breaking social norms. This might not necessarily cause a problem; the study by McGrath et al. (2018) also demonstrated an association between giving testimonials and both reduced gambling severity and overall satisfaction with the GA programme. Thus, individuals should still derive benefit from the meetings, even if they decide not to listen to others’ testimonials. On the other hand, this may be concerning since social support variables have been found to consistently mediate the groups impact on abstinence, at least in Alcoholics Anonymous (Groh et al., 2008); non-compliance with social norms might jeopardize these social networks, in turn having an impact on abstinence. Thus, the results of this study imply a complex situation whereby there is a tension between the individual versus the group.

There is a scarcity of research into Gamblers Anonymous, and more broadly a scarcity of research into the underlying mechanisms involved in mutual aid groups. The literature suggests that mutual aid groups fulfil their function through positive social networks and group cohesion. The results of this study suggest that perhaps the social networks created are dynamic and changeable given different scenarios. Future research could consider other ways GA groups (or mutual aid groups in general) could be affected, or indeed, how they could adapt to new scenarios. This study therefore provides insights into the experience of attending GA meetings during the COVID-19 pandemic, highlights the complex interplay between self versus other in the context of GA meetings and demonstrates the effect of Zoom on these identities.

### Limitations

This study does however present several limitations. Primarily, those individuals who agreed to be interviewed may not reflect the experiences of those not captured by this study. Indeed, given the high drop-out rates described by the participants in this study, the results do not include important information about, for example, why those people who ceased attending did so. Future research could explore this area. Secondly, whilst every effort was made to recruit a diverse sample, the majority of participants were white British males which could have limited responses. Future research should focus on aiming to capture the experiences of a more ethnically and gender diverse range of participants.

Inevitably, there is a degree of subjectivity involved in qualitative analysis. Throughout the study, the researcher was mindful of their own personal biases and idiosyncrasies, which can influence qualitative research. In this case, the researcher has experience of a problem gambler in their personal life, however, it can be argued that rather than hindering the research process, or skewing it in some way, instead it allowed the researcher to connect with participants deeply through empathy and understanding.

Given that at the core of Gamblers Anonymous is anonymity and trust - which is ensured through members being exclusive to problem gamblers - the researcher’s status as a non-gambler/GA member may have affected participant responses. The results of this study demonstrate complex social structures which were of great importance to participants. Thus, participants may have perceived the researcher as an ‘outsider’ or not felt comfortable revealing information about their groups. To try and mitigate this, the researcher was mindful to maintain unconditional acceptance, staying open and non-judgmental throughout the interviews.

Whilst a single researcher conducted, transcribed, and coded the interviews, the analytic process involved repeated discussions with the research team to ensure the analysis stayed as close to the data as possible. Furthermore, it is hoped that this limitation is managed, in part, by explicit presentation of the researchers’ own theoretical commitments, and by the transparency of the analytic process.

## Conclusion

Despite being the most widely accessed form of support for problem gamblers, Gamblers Anonymous has been largely overlooked in academic research. Furthermore, to date no research has explored how mutual aid groups like Gamblers Anonymous have been affected by the COVID-19 pandemic. The current study addressed this gap in the literature by providing an insight into members’ experiences of attending GA meetings and how they feel this has been impacted upon by the COVID-19 pandemic and the move to online meetings.

The results revealed three main themes: (1) ‘practicalities of GA in lockdown’, which described practical benefits such as more opportunity to attend different meetings which in turn expanded participants’ perspectives and social networks; (2) ‘the importance of relationships in GA’, which described strong and enduring social networks that were created, maintained, and strengthened by feelings of solidarity; and (3) ‘therapeutic elements of the meetings’, such as psychological contract making which helped participants to stay abstinent. There was also a transcending theme of ‘tension between individual versus group identity’.

Whilst the meetings offered a lifeline in an otherwise precarious time and provided several practical benefits, the move to online meetings was seen as having disrupted the social elements of the group. They therefore were not able to completely replicate the physical meetings, ultimately leading to group members behaving more individualistically.

The literature suggests that mutual aid groups fulfil their function through positive social networks and group cohesion (Groh et al., 2008). This study has highlighted a complex interplay between self versus other in the context of GA meetings and demonstrates the effect of Zoom on these identities; the results of this study suggest that perhaps the social networks created are actually dynamic and changeable given different scenarios, are not always the driving force behind the positive effects of these groups.

Therefore, whilst still providing a lifeline during COVID-10, the online GA meetings were not able to completely sufficiently replicate the value individuals gained from the physical meetings. The transition also resulted in disruptions to group dynamics - and individual interactions within each group - which ultimately lead to group members behaving more individualistically, than in face-to-face meetings. These results could help Gamblers Anonymous and other service providers to make more informed choices about how to support their users, should we face another global crisis. This knowledge will also help GA, other mutual aid groups and other service providers to understand their members’ needs, ultimately optimizing the support they can offer.

## Data Availability

The datasets generated during and/or analysed during the current study are available from the corresponding author on reasonable request.
